# Modulation of circular current and associated magnetic field in a molecular junction: A new approach

**DOI:** 10.1038/srep43343

**Published:** 2017-03-03

**Authors:** Moumita Patra, Santanu K. Maiti

**Affiliations:** 1 Physics and Applied Mathematics Unit, Indian Statistical Institute, 203 Barrackpore Trunk Road, Kolkata-700 108, India

## Abstract

A new proposal is given to control local magnetic field in a molecular junction. In presence of finite bias a net circular current is established in the molecular ring which induces a magnetic field at its centre. Allowing a direct coupling between two electrodes, due to their close proximity, and changing its strength we can regulate circular current as well as magnetic field for a wide range, without disturbing any other physical parameters. We strongly believe that our proposal is quite robust compared to existing approaches of controlling local magnetic field and can be verified experimentally.

The study of electronic transport through single molecules has been the objects of intense research due to the fact that molecular components can be utilized as significant functional elements in electronic devices. In 1974 Aviram and Ratner^1^ first proposed a unimolecular device considering a molecule as the basic building block, and latter many works have been done^2^,^3^,^4^,^5^,^6^,^7^,^8^,^9^,^10^,^11^,^12^,^13^,^14^,^15^,^16^,^17^ to explore electron transport through different simple as well as complex molecular structures.

Though a wealth of literature knowledge has been established in the field of molecular transport, most of the works have focused essentially on net junction current, while very few attempts have been made^18^,^19^,^20^,^21^,^22^,^23^,^24^,^25^,^26^ so far where distribution of current in different arms of a molecular junction has been analyzed. In presence of finite bias a net circular current is established in the molecular ring which results a non-zero magnetic field at its centre. Depending on bias voltage and molecule-to-electrode interface geometry this magnetic field becomes quite high and in some cases it becomes ~Millitesla (mT) or even ~T^24^,^25^. A number of recent investigations of electronic transport through molecular junctions^24^,^25^,^27^,^28^,^29^ have shown that in the limit of weak molecule-to-electrode coupling much higher circular current is obtained in molecular loops compared to the net transport current across the junction. Possible applications of such high *local* magnetic fields in molecular systems came into limelight following the realization of controlling spin orientation^20^ of a cation site embedded in a conducting junction by the local magnetic field induced by loop current or the prediction of carbon nanotubes as molecular solenoids^29^,^30^,^31^. Considering a T-shape tape-porphyrin molecular wire Tagami and Tsukada have shown that the current which is established in the molecular loop produces the local magnetic field ~0.1 T at the bias voltage of 1.2 V, that can be utilized to regulate local spin orientation, and it has an important viewpoint as detecting the spin orientation by means of changing the bias polarity one can get a clear idea of the existence of circular current in the molecular loop. The phenomenon of circular current is also directly linked with other context the so-called current transfer process^32^, where a current imposed in one path affects a current in other arms exploring the quantum interference affect, which certainly demands a detailed analysis.

Though several suggestions were made for the possible exploitations of such high local magnetic fields at the molecular regime, probably the most significant application can be the generation of spin-based quantum computers^33^,^34^,^35^,^36^. To achieve this goal proper spin regulation is highly important, which on the other hand requires finite tuning of magnetic field in a localized region. Few propositions have already been done along this direction. For instance, using phase locked infra-red laser pulses Pershin and Piermarocchi have shown^37^ that circular current can be established in an isolated quantum ring where the magnetic field reaches up to few mT. Utilizing this local magnetic field they have shown how the spin orientation, provided e.g., by a magnetic impurity embedded at the ring centre or on top of a ring, can be locally controlled by magnetic field due to the current in the ring. In other work Lidar and Thywissen have established^38^ that a localized magnetic field, which may reach up to 10 mT, can be generated with the help of an infinite array of parallel current carrying wires, though it has severe limitation due to heating effect and one has to work at much lower temperature (<2.4 mK). Comparing all these propositions we can argue that bias induced magnetic field, associated with circular current, in a nano-junction is quite robust and easy to operate^24^,^25^,^39^,^40^,^41^. The essential motivations behind the consideration of a molecular junction with loop structure(s) are as follows: (a) Bias induced circular current produces strong magnetic field (that can also be varied in a wide range) at the molecular/nano-scale level compared to the net junction current. At this length scale simple quantum wire cannot produce such a strong magnetic field. (b) Exploiting quantum interference effect several anomalous features can be observed in ring-like geometry, which are not possible in conducting junctions without any loop. (c) Spectral response of magnetic ions placed near or on the molecular ring to the current induced magnetic field gives an atypical observation of magnetic shielding and deshielding effect in NMR spectra of aromatic molecules^42^. (d) Another operation can also be implemented by assigning up and down spin states as two binary logic bits 0 and 1. The flipping of spin states, as a result of local magnetic field will correspond to the switching between 0 and 1 states, which thus carry quantum information. This is the basic principle used in designing quantum computation which reduces much power dissipation, compared to the conventional computing which is charge based where two different charges are assigned to encode binary logic bits 0 and 1, and involves charge flow that costs excessive power loss. Thus, the study of circular current due to voltage bias in the molecular scale level is certainly worthy and interesting.

In the present paper we essentially focus on how to control circular current and associated magnetic field in a molecular junction having single or multiple loops coupled to source and drain electrodes. Due to close proximity electrons can directly hop between the end atomic sites of these two electrodes and tuning this coupling strength, which is done simply by changing the orientation/position of these electrodes, we can regulate circular current and associated magnetic field in a wide range. No one has addressed this issue before, to the best of our concern, and certainly gives a new insight to modulate electron transmission through a nano-junction.

## Molecular Model and Theoretical Framework

**Figure 1:**
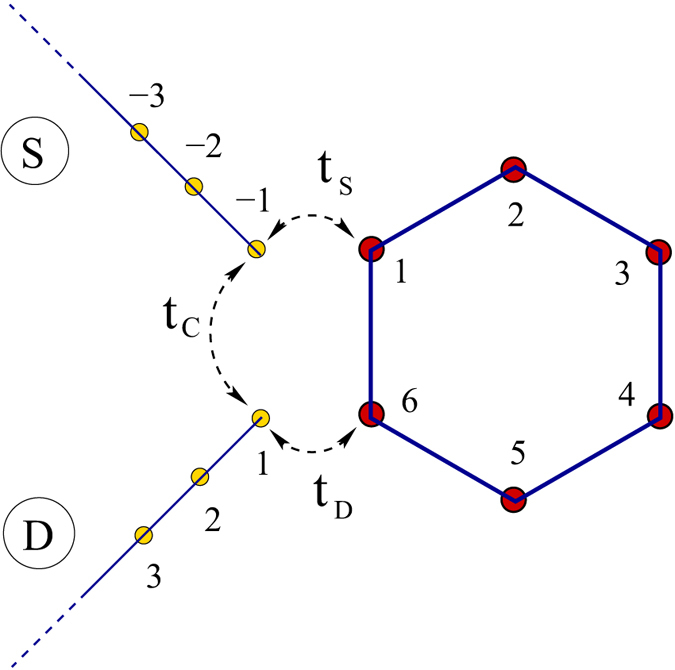
Schematic view of a molecular junction where a benzene molecule is coupled to source (S) and drain (D) electrodes in ortho-configuration. Due to close proximity there exists a *direct coupling* between S and D which provides a *new path* along with the conventional path i.e., the bridging molecule.

The molecular junction is schematically shown in Fig. 1 where a benzene molecule is coupled to two one-dimensional (1D) perfect electrodes, viz, source and drain. The electrodes are connected to the molecule in ortho-configuration such that an electron can directly hop between the end atomic sites of these electrodes due to their close proximity which essentially provides a *new path* in addition to the conventional path i.e., the molecular ring.

To describe this model we use tight-binding (TB) framework, which is most convenient for analyzing electron transport through a molecular junction particularly in the limit of non-interacting electrons. The full Hamiltonian of the molecular junction can be written as: *H* = *H*_*M*_ + *H*_*S*_ + *H*_*D*_ + *H*_*T*_, where *H*_*M*_ corresponds to the Hamiltonian for the molecule, *H*_*S(D*)_ represents the Hamiltonian for the source(drain) electrode and *H*_*T*_ gives the tunneling Hamiltonian. In TB framework these sub-Hamiltonians are expressed as follows:


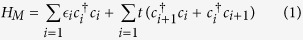



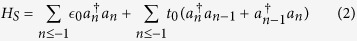



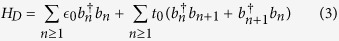



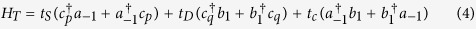


here 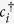 and *c*_*i*_ correspond to the creation and annihilation operators, respectively, for an electron at *i*-th site of the molecular ring, while these operators are 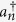, *a*_*n*_ and 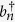, *b*_*n*_ for the source and drain electrodes, respectively. The molecule is characterized by the on-site potential 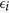 and nearest-neighbor hopping integral *t*, whereas for the side attached electrodes these parameters are 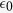 and *t*_0_, respectively. *t*_*S*_ describes the molecular coupling with the source and it is *t*_*D*_ for the drain. These electrodes are connected at the sites *p* and *q* (which are variable and nearest-neighbors). For this molecular junction (Fig. 1) *p* = 1 and *q* = 6. *t*_*c*_ represents the inter-electrode coupling and it can be tuned either by changing the separation between the electrodes or by rotating them. Our main concern in this article is how *t*_*c*_ affects electronic transmission through the molecular junction.

To evaluate transmission probability across the molecular wire we adopt wave-guide theory^24^,^25^,^43^,^44^,^45^ where a set of coupled linear equations involving wave amplitudes at different lattice sites are solved. These coupled equations are generated from the Schrödinger equation 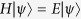, considering 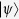 in the form:


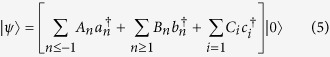


where *A*_*n*_, *B*_*n*_ and *C*_*i*_ correspond to the amplitudes for an electron at site *n* of the source/drain electrode and at the site *i* of the ring, respectively. In terms of the reflection and transmission coefficients *r* and *τ*, the amplitudes *A*_*n*_ and *B*_*n*_ can be written as 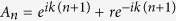 and *B*_*n*_ = *τe*^*ikn*^, where we assume that a plane wave with unit amplitude is coming from the source. Thus for each wave vector *k*, associated with energy *E*, we calculate *τ* from the set of linear equations and get the transmission probability


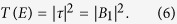


Using the transmission function *T(E*), net junction current at absolute zero temperature for a particular voltage bias *V* is determined from the relation^46^


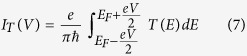


where *E*_*F*_ is the equilibrium Fermi energy.

Now to find circular current in the molecular ring we need to calculate current carried by individual bonds. For any such bond, connecting the sites *i* and *i* + 1, it becomes^24^,^25^


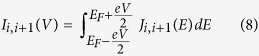


where 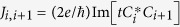. With these bond currents the net circular current is calculated from the relation^24^,^25^,^39^,^40^,^41^


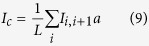


where *L* = *Na, a* being the lattice spacing and *N* represents the total number of atomic sites in the bridging molecule. We assign a positive sign to a current flowing in the anti-clockwise direction.

Due to this circular current a net magnetic field is established. The local magnetic field at any arbitrary point *r* (say) inside the molecule can be determined using the Biot-Savart’s law^24^,^25^,^39^,^40^,^41^


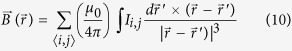


where *μ*_0_ is the magnetic constant.

## Results and Discussion

Based on the above theoretical framework now we present our results which include two-terminal transmission probability, junction current, circular current and associated magnetic field at the ring centre. There are some physical parameters those values are kept constant throughout the numerical calculations. These parameter are described as follows. In the molecular ring we choose 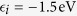 and *t* = 2.5 eV, while for the side-attached electrodes they are: 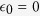 and *t*_0_ = 3 eV. The molecule-to-electrode coupling parameters (*t*_*S*_ and *t*_*D*_) are fixed at 1 eV, and the equilibrium Fermi energy *E*_*F*_ is set at zero. The values of other physical parameters, those are not same for all figures, are specified in appropriate places. To calculate magnetic field we assume the perpendicular distance from the centre of the benzene ring to any *C*-*C* bond is ~0.13 nm^25^.

**Figure 2:**
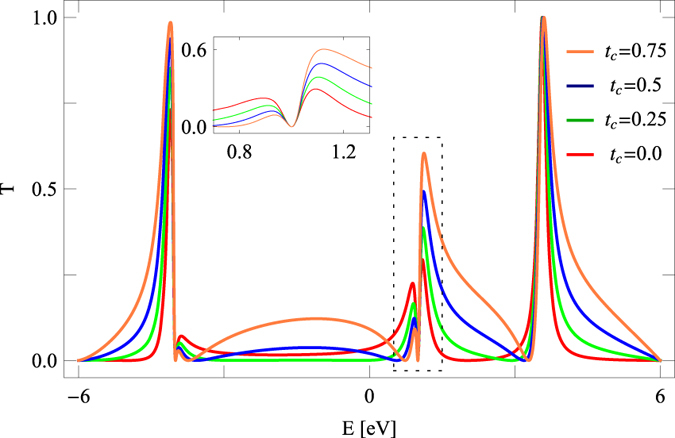
Two-terminal transmission probability (*T*) as a function of energy (*E*) for the molecular junction (shown in Fig. 1) at some typical values of *t*_*c*_. The inset of the figure represents the zoomed version of the dashed framed region. A sharp dip (vanishing transmission) is observed at ~*E* = 1 eV and across this dip resonant peaks exhibit completely opposite scenario. In the right side the peak height gradually increases with *t*_*c*_, while it decreases with *t*_*c*_ in the other side of the dip. The similar feature is also observed in other energies where a transmission peak is followed by a dip.

Before going to the central part of our analysis i.e., how direct coupling (*t*_*c*_) affects circular current and induced magnetic field, let us focus on transmission probability and junction current. In Fig. 2 we present the variation of two-terminal transmission probability as a function of injecting electron energy for the benzene molecule considering some typical values of *t*_*c*_. For *t*_*c*_ = 0 fine resonant peaks associated with energy eigenvalues of the molecular ring are obtained while the transmission probability drops very close to zero for all other energies. This behavior has already been discussed in several earlier papers^12^,^17^,^24^ for ortho-connected benzene ring. The situation becomes very interesting when we include the effect of *t*_*c*_. Apparently it shows that electron gets transmitted almost for the entire energy window and the transmission amplitude gradually increases with the rise of *t*_*c*_. But, a careful inspection yields many fascinating points. To reveal this fact we select a small part of the spectrum, the dashed framed region, and place its zoomed version in the inset. Very interestingly we see that a sharp dip (vanishing transmission) appears at ~*E* = 1 eV, and above and below this dip resonant curves exhibit completely opposite behavior. One side of this dip, the height of the peak increases while in the other side it gradually decreases with respect to the coupling parameter *t*_*c*_. This feature is also observed in other energies where a transmission peak is followed by a dip. It is an important observation since one can get higher and/or lower electronic transmission at different energies simply by tuning the external coupling parameter *t*_*c*_, without changing any other physical variables. The anomalous feature in this ring-like geometry is observed due to the presence of the *new path* between the electrodes. A combined interference effect among electronic waves passing through different arms (upper and lower arms of the molecular ring including the external new path) leads to such a nice phenomenon, and of course would not be noticed in molecular junctions without any loop structure. Thus a competition takes place between the interfering paths i.e., the molecular arms and the external path, and the response depends on the resultant of all these paths. For strong enough *t*_*c*_ electrons mostly follow the external path, avoiding the conventional molecular ring.

**Figure 3:**
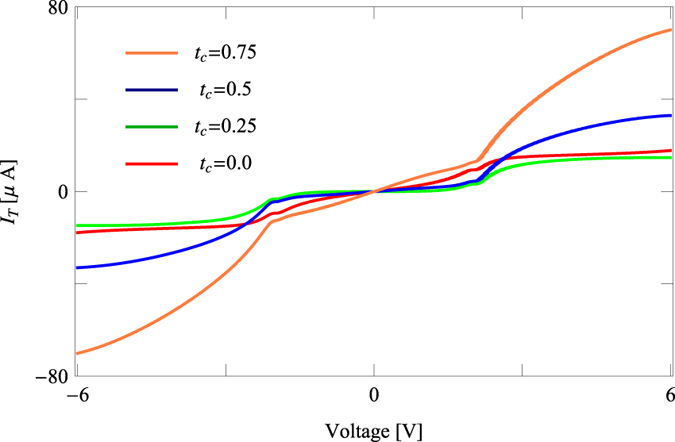
Junction current *I*_*T*_ as a function of applied bias voltage *V* for the ortho-connected benzene molecule considering identical values of *t*_*c*_ as taken in Fig. 2.

The above signature is clearly reflected in the current-voltage characteristics as the junction current is evaluated by integrating transmission function *T* over an energy window associated with the bias voltage *V* (Eq. 7). Figure 3 displays the dependence of junction current *I*_*T*_ with applied bias voltage *V* for the othro-connected benzene molecule for some specific values of *t*_*c*_. The current starts increasing approximately linearly with *V*, while a sudden change of its amplitude takes place at a critical voltage (*V* ~ 2 V). This sudden jump is associated with the crossing of one of the resonant energy levels which is clearly seen from the *T*-*E* spectrum (Fig. 2). Most interestingly we see that for low enough *t*_*c*_ current is smaller (green line) compared to the molecular junction without any *t*_*c*_ (red line), but eventually the current increases sharply with *t*_*c*_, following the *T*-*E* curve (Fig. 2).

**Figure 4:**
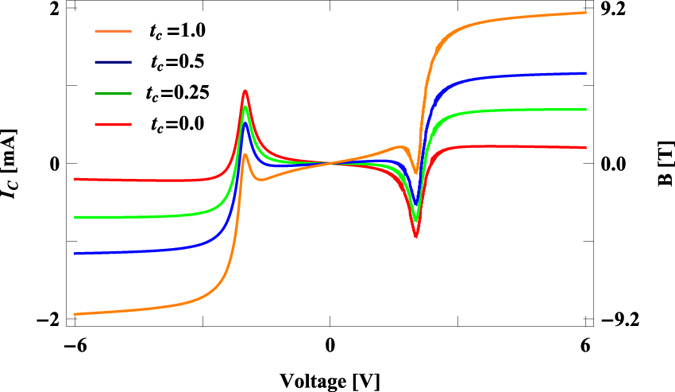
Circular current *I*_*c*_ and associated magnetic field *B* at the centre of the benzene molecule as a function of bias voltage for different values of *t*_*c*_.

Now we concentrate on the variations of circular current and induced magnetic field produced at the ring centre for the molecular junction given in Fig. 1. The results are presented in Fig. 4. Unlike junction current (*I*_*T*_), circular current (*I*_*c*_) changes its sign in different voltage regimes for any side (positive or negative) of the applied bias. And also the magnitude of *I*_*c*_ may be sufficiently large compared to the transport current *I*_*T*_, depending on the external voltage *V*. For narrow voltages when no resonant energy level appears within the voltage window we get vanishing circular current. While a non-zero contribution comes when anyone of such energy levels lies within the voltage window. With increasing voltage more and more resonant energy levels appear within the window and all of them contribute to the current, resulting a net circular current which may be positive or negative depending of the sign of the dominating energy levels (the sign reversal can be clearly understood from the forthcoming analysis). Though junction current always increases with voltage bias in conventional conducting junctions (where negative differential resistance effect is not considered). Most importantly, the magnetic field which is developed at the ring centre as a result of this circular current is surprisingly high, and it increases significantly with *t*_*c*_. For a wide voltage region (>~2 V) the magnetic field remains almost constant for any specific *t*_*c*_ (Fig. 4), following *I*_*c*_, as within this window there is no other energy channel to contribute current.

**Figure 5:**
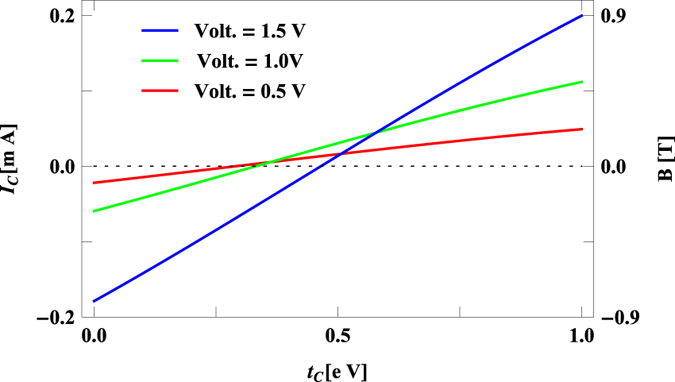
Dependence of *I*_*c*_ and *B* with *t*_*c*_ for the benzene molecule at some typical bias voltages. The dashed horizontal line represents the line of zero circular current.

In order to see more clearly the dependence of circular current and associated magnetic field on *t*_*c*_ in Fig. 5 we present their variations as a function of *t*_*c*_ for some typical values of bias voltage *V*. It is observed that both the circular current and induced magnetic field decrease with *t*_*c*_ and reaching to zero, and eventually they increase with increasing the coupling parameter *t*_*c*_. For lower *t*_*c*_, one of the doubly degenerate energy levels comes within the voltage window (the degeneracy disappears as a result of molecule-to-electrode coupling) which contributes to the current. But as we increase *t*_*c*_ the other resonant energy levels also appear within this bias window and contributes current in opposite direction with respect to the earlier one yielding a reduction of current. Finally, when they become exactly opposite with each other a vanishing net current is obtained. Beyond this critical value of *t*_*c*_ both these states contribute in the same direction providing a resultant higher circular current. From this behavior it can be manifested that tuning the coupling between source and drain electrodes one can regulate circular current and thus locally control induced magnetic field for a wide range starting from zero to few Tesla. Certainly this phenomenon gives a new way of controlling magnetic field in a specific region without disturbing any physical parameters of the system and can be utilized in designing effective spin based quantum devices.

**Figure 6:**
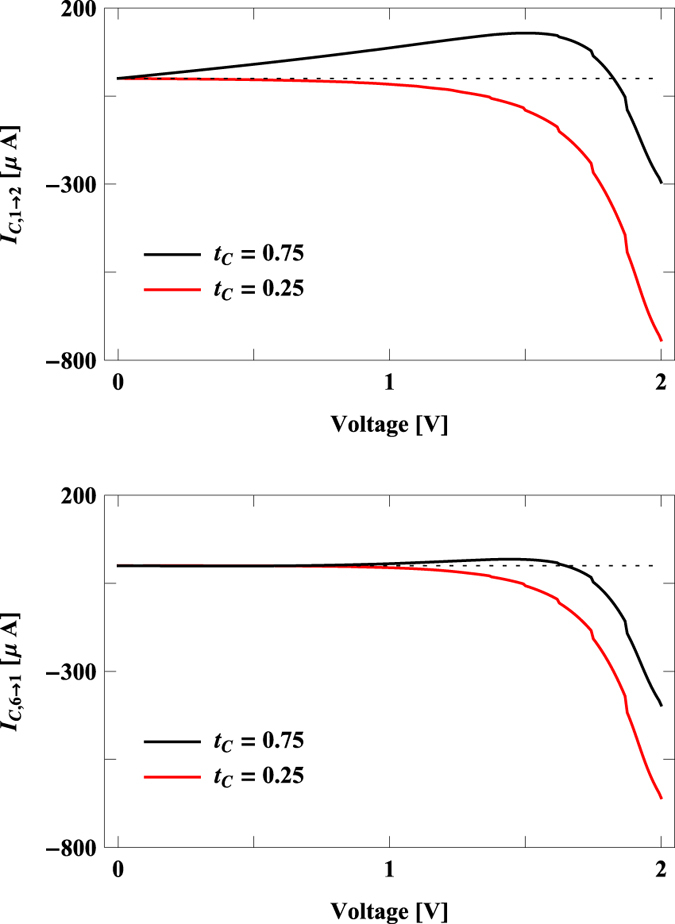
Bond currents (two bonds are taken from two arms of the molecular junction) as a function of bias voltage for the benzene molecule at two different values of *t*_*c*_. We choose these two typical values of *t*_*c*_ to explore the sign reversal of circular current and induced magnetic field displayed in Fig. 5. The dashed horizontal line represents the line of zero bond current.

The sign reversal of circular current, and hence the induced magnetic field, with *t*_*c*_ for different voltages can be clearly understood from the variation of bond currents, as circular current is determined from the bond currents (Eq. 9). The variations of two bond currents, where the bonds are chosen from the two arms of the junction, with bias voltage are shown in Fig. 6. The results are computed for two typical values of *t*_*c*_, one for which *I*_*c*_ is negative in Fig. 5 (*t*_*c*_ = 0.25 eV) and for the other (*t*_*c*_ = 0.75 eV) *I*_*c*_ becomes positive in Fig. 5. From the spectra given in Fig. 6 it is clearly noticed that for a fixed *t*_*c*_ bond current changes its sign with voltage. At the same footing for a fixed bias voltage the sign reversal of bond current also takes place with the change of *t*_*c*_ yielding a change of sign of the circular current *I*_*c*_.

**Figure 7:**
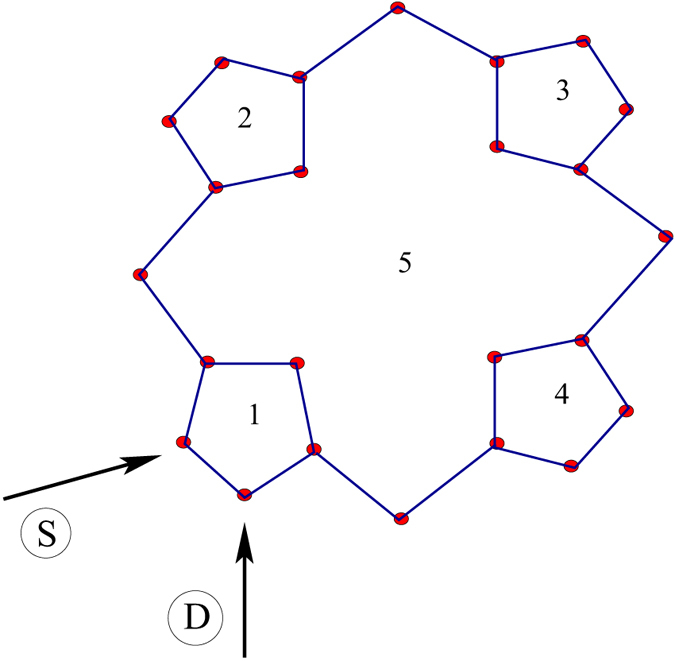
Schematic view of another molecular junction where the benzene molecule is replaced by porphyrin molecule. The ring-to-electrode configuration remains same as in Fig. 1. The numbers 1, 2 …, 5 represents the loop numbers.

**Figure 8:**
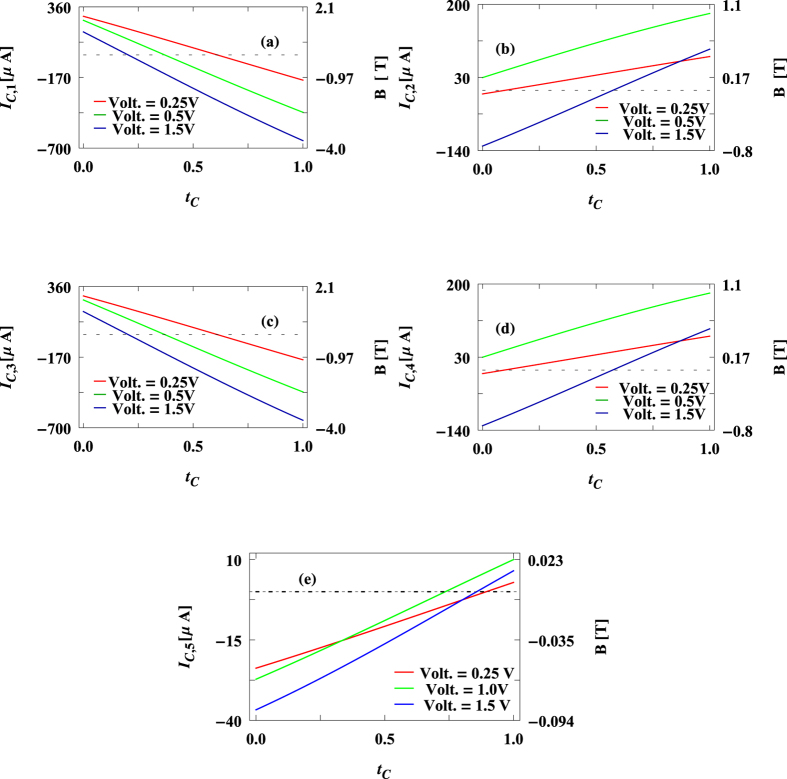
Circular current (*I*_*c,n*_, *n* = 1, 2 …, 5) and induced magnetic field (*B*) in different sub-loops (presented in (**a**–**e**)) of the molecular junction shown in Fig. 7 as a function of *t*_*c*_ for some typical bias voltages.

The results presented above are worked out for the molecular wire containing only the benzene molecule. So the question naturally comes whether similar kind of behavior is observed in other molecular wires with higher number of loops. To answer it now we analyze the behavior of circular current and associated magnetic field of other relevant molecular structure, namely, porphyrin, that is connected with the source and drain electrodes as prescribed in Fig. 7, analogous to the configuration given in Fig. 1. The results are shown in Fig. 8. Qualitatively the circular currents and induced magnetic fields in different sub-loops of the porphyrin molecule exhibit almost similar characteristic features to what we get for the case of benzene molecule (Fig. 5). An additional important feature is that in some wide voltage regions circular currents in different loops are opposite in sign. Note that the magnitude of circular current in the four outer loops is much larger compared to the bigger inner one. Here it is relevant to note that based on circular current induced magnetic field, controlling of spin orientation of a cation site embedded in the T-shape tape-porphyrin molecular wires has already been discussed elaborately in ref. 20. Identical features are also obtained for other molecular junctions involving several such molecular loops (not shown here) which we confirm through our detailed numerical calculations. Thus, one can in principle consider a molecular system where magnetic fields of variable strengths can be established in different sub-regions of the geometry that might be very helpful for designing nanoelectronic quantum devices.

## Summary

In this work we have demonstrated how to control local magnetic field in a wide region (from zero to a surprisingly large value) considering a simple molecular structure by introducing a new path between two electrodes. Using the wave-guide theory, we have calculated two-terminal transmission probability, junction current, circular current and current induced magnetic field at ring centre(s) based on a coherent tight-binding framework. Our finding, to the best of our concern, gives a unique idea of regulating electron transport through a conducting junction.

## Additional Information

**How to cite this article**: Patra, M. and Maiti, S. K. Modulation of circular current and associated magnetic field in a molecular junction: A new approach. *Sci. Rep.* **7**, 43343; doi: 10.1038/srep43343 (2017).

**Publisher's note:** Springer Nature remains neutral with regard to jurisdictional claims in published maps and institutional affiliations.
